# Supplementary material for: Comparative physiological and transcriptomic analyses reveal the mechanisms of CO2 enrichment in promoting the growth and quality in Lactuca sativa
Source: PLoS One. 2023 Feb 3;18(2):e0278159. doi: 10.1371/journal.pone.0278159 (PMC9897578; doi:10.1371/journal.pone.0278159)
Supplement: S1 Fig — (PDF) [file pone.0278159.s001.pdf]

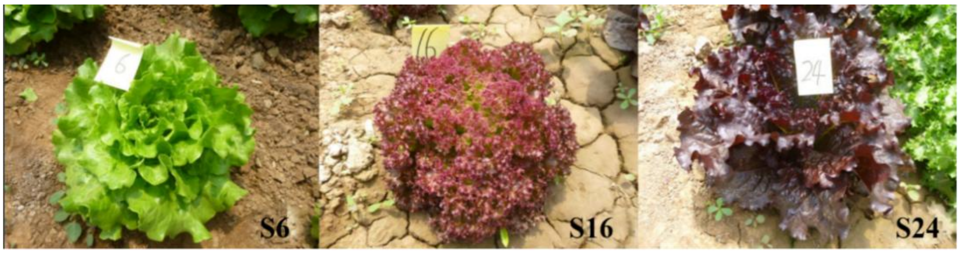

S1 Fig. Materials for testing (Three materials respectively represent 3 colors, S6 represents green, S16 represents green and purple, and S24 represents purple lettuce)
