# Supplementary material for: Comparative physiological and transcriptomic analyses reveal the mechanisms of CO2 enrichment in promoting the growth and quality in Lactuca sativa
Source: PLoS One. 2023 Feb 3;18(2):e0278159. doi: 10.1371/journal.pone.0278159 (PMC9897578; doi:10.1371/journal.pone.0278159)
Supplement: S1 Table — (PDF) [file pone.0278159.s002.pdf]

S1 Table. The primer sequences for qRT-PCR

Gene ID	Forward primer	Reverse primer
ubiquitin	GAAGAAGACCTACACCAAGCCAAAG	ACTCAGCATTAGGGCACTCTTTCC
Lsat_1_v5_gn_4_142020	GTCACTGGGTACGTCATCGTT	TTGAATGCGTCTTCGTCCGT
Lsat_1_v5_gn_4_182521	ACGAAAAAGCACACAAGAGACG	AGCAATGGCCTCCTCATACG
Lsat_1_v5_gn_5_139561	TTTTACGCCGGACAGGTGAT	AGTTTGGGTCTTCTGCGTGG
Lsat_1_v5_gn_5_136060	CACCTTCACCACTCACGCAG	AGCGGCTTTGATGATGTTGG
Lsat_1_v5_gn_5_141001	CAGGCGTTTGTTAGCTTCGG	AGACCCACCAAGAACACCCT
Lsat_1_v5_gn_6_26160	GACGCTGTGAAGTCTCCCAA	CATCGCCTCCGACTCCTTAC
Lsat_1_v5_gn_7_73881	AGGCGGTTGCTCGGTTTTT	AGCAATTCTGTTCCTCGACAA
Lsat_1_v5_gn_9_23900	GACATGGGTCTGGAAGCGTAT	GGGCGATAAGGCTGTTGATG
