# Supplementary material for: Comparative physiological and transcriptomic analyses reveal the mechanisms of CO2 enrichment in promoting the growth and quality in Lactuca sativa
Source: PLoS One. 2023 Feb 3;18(2):e0278159. doi: 10.1371/journal.pone.0278159 (PMC9897578; doi:10.1371/journal.pone.0278159)
Supplement: S2 Table — (PDF) [file pone.0278159.s003.pdf]

S2 Table. Sample sequencing data evaluation statistics

sample	Clean bases	GC Content	≥Q30%	Clean reads	Mapped reads	Unique mapped reads	Multiple mapped reads
SCD6	7,079,690,440	44.54%	89.42%	43,870,658	35,604,822 (81.16%)	34,387,332 (78.38%)	1,217,490 (2.78%)
SCDB16	6,859,457,176	44.00%	90.42%	45,869,962	29,879,113 (65.14%)	29,062,531 (63.36%)	816,582 (1.78%)
SCD24	7,079,690,440	45.03%	89.35%	47,430,668	37,451,608 (78.96%)	35,781,079 (75.44%)	1,670,529 (3.52%)
SCF6	7,738,545,054	44.54%	89.93%	51,720,602	42,775,606 (82.71%)	41,269,156 (79.79%)	1,506,450 (2.91%)
SCFB16	6,235,826,904	44.33%	90.04%	41,722,962	31,930,909 (76.53%)	30,964,475 (74.21%)	966,434 (2.32%)
SCF24	8,540,792,454	45.56%	88.87%	57,210,692	45,097,111 (78.83%)	42,709,742 (74.65%)	2,387,369 (4.17%)

Note: Clean bases: total bases of high-quality reads; GC content: percentage of total bases in high-quality reads G/C bases; ≥Q30%: quality value greater than or equal to 30 reads Percentage of total bases. Mapped Reads: the reads compared to the reference genome and the percentage of Clean Reads; Unique Mapped Reads: the number of reads uniquely compared to the reference genome; Multiple Map Reads: the number of reads multiplexed to the reference genome.
