# Supplementary material for: Comparative physiological and transcriptomic analyses reveal the mechanisms of CO2 enrichment in promoting the growth and quality in Lactuca sativa
Source: PLoS One. 2023 Feb 3;18(2):e0278159. doi: 10.1371/journal.pone.0278159 (PMC9897578; doi:10.1371/journal.pone.0278159)
Supplement: S4 Table — (PDF) [file pone.0278159.s005.pdf]

S4 Table. KEGG Significant Enrichment of different expression genes

#Kegg_pathway	ko_id	Cluster_frequency	Genome_frequency	P-value	Corrected_P-value	rich_factor
Limonene and pinene degradation	ko00903	2 out of 41 4.8780487804878%	11 out of 7260 0.151515151515152%	0.001657241	0.056346202	32.19512195
Starch and sucrose metabolism	ko00500	6 out of 41 14.6341463414634%	289 out of 7260 3.98071625344353%	0.005245023	0.178330798	3.6762596
Plant hormone signal transduction	ko04075	7 out of 41 17.0731707317073%	391 out of 7260 5.38567493112948%	0.0057086	0.194092399	3.170107916
Biosynthesis of unsaturated fatty acids	ko01040	3 out of 41 7.31707317073171%	86 out of 7260 1.18457300275482%	0.012371648	0.420636036	6.176971072
Galactose metabolism	ko00052	3 out of 41 7.31707317073171%	104 out of 7260 1.43250688705234%	0.020531342	0.698065619	5.107879925
Stilbenoid, diarylheptanoid and gingerol biosynthesis	ko00945	2 out of 41 4.8780487804878%	44 out of 7260 0.606060606060606%	0.025347632	0.861819473	8.048780488
Regulation of autophagy	ko04140	2 out of 41 4.8780487804878%	50 out of 7260 0.68870523415978%	0.032135134	1	7.082926829
Photosynthesis - antenna proteins	ko00196	1 out of 41 2.4390243902439%	30 out of 7260 0.413223140495868%	0.156540098	1	5.902439024
Porphyrin and chlorophyll metabolism	ko00860	2 out of 41 4.8780487804878%	66 out of 7260 0.909090909090909%	0.053190534	1	5.365853659
Cyanoamino acid metabolism	ko00460	2 out of 41 4.8780487804878%	69 out of 7260 0.950413223140496%	0.057566507	1	5.132555673
One carbon pool by folate	ko00670	1 out of 41 2.4390243902439%	36 out of 7260 0.495867768595041%	0.184844585	1	4.918699187
Diterpenoid biosynthesis	ko00904	1 out of 41 2.4390243902439%	36 out of 7260 0.495867768595041%	0.184844585	1	4.918699187
Other glycan degradation	ko00511	1 out of 41 2.4390243902439%	39 out of 7260 0.537190082644628%	0.198647169	1	4.540337711
Pentose and glucuronate interconversions	ko00040	3 out of 41 7.31707317073171%	122 out of 7260 1.68044077134986%	0.031052289	1	4.354258297
Sulfur metabolism	ko00920	1 out of 41 2.4390243902439%	42 out of 7260 0.578512396694215%	0.212221647	1	4.216027875
Fatty acid elongation	ko00062	1 out of 41 2.4390243902439%	44 out of 7260 0.606060606060606%	0.221146392	1	4.024390244
Nitrogen metabolism	ko00910	1 out of 41 2.4390243902439%	53 out of 7260 0.730027548209366%	0.260101967	1	3.341003221
beta-Alanine metabolism	ko00410	1 out of 41 2.4390243902439%	61 out of 7260 0.840220385674931%	0.293128508	1	2.902838864
Ubiquinone and other terpenoid-quinone biosynthesis	ko00130	1 out of 41 2.4390243902439%	61 out of 7260 0.840220385674931%	0.293128508	1	2.902838864
Tropane, piperidine and pyridine alkaloid biosynthesis	ko00960	1 out of 41 2.4390243902439%	63 out of 7260 0.867768595041322%	0.301157736	1	2.81068525
Phenylalanine, tyrosine and tryptophan biosynthesis	ko00400	1 out of 41 2.4390243902439%	65 out of 7260 0.895316804407714%	0.30909796	1	2.724202627
Fatty acid metabolism	ko01212	3 out of 41 7.31707317073171%	200 out of 7260 2.75482093663912%	0.102527961	1	2.656097561
RNA degradation	ko03018	3 out of 41 7.31707317073171%	215 out of 7260 2.96143250688705%	0.120472306	1	2.470788429
Circadian rhythm - plant	ko04712	1 out of 41 2.4390243902439%	72 out of 7260 0.991735537190083%	0.33620165	1	2.459349593
Amino sugar and nucleotide sugar metabolism	ko00520	2 out of 41 4.8780487804878%	184 out of 7260 2.53443526170799%	0.278808367	1	1.924708378
Fructose and mannose metabolism	ko00051	1 out of 41 2.4390243902439%	93 out of 7260 1.28099173553719%	0.411436631	1	1.904012589
Arginine and proline metabolism	ko00330	1 out of 41 2.4390243902439%	100 out of 7260 1.37741046831956%	0.434614329	1	1.770731707
Glyoxylate and dicarboxylate metabolism	ko00630	1 out of 41 2.4390243902439%	111 out of 7260 1.52892561983471%	0.469248485	1	1.59525379
Glycine, serine and threonine metabolism	ko00260	1 out of 41 2.4390243902439%	121 out of 7260 1.66666666666667%	0.498931985	1	1.463414634
Biosynthesis of amino acids	ko01230	2 out of 41 4.8780487804878%	370 out of 7260 5.09641873278237%	0.625829867	1	0.957152274
Carbon metabolism	ko01200	2 out of 41 4.8780487804878%	427 out of 7260 5.88154269972452%	0.704107255	1	0.829382533
Glycolysis / Gluconeogenesis	ko00010	1 out of 41 2.4390243902439%	216 out of 7260 2.97520661157025%	0.71114365	1	0.819783198
Phenylpropanoid biosynthesis	ko00940	1 out of 41 2.4390243902439%	233 out of 7260 3.20936639118457%	0.738462484	1	0.75997069
Protein processing in endoplasmic reticulum	ko04141	1 out of 41 2.4390243902439%	425 out of 7260 5.85399449035813%	0.916282549	1	0.416642755
