# Supplementary material for: Comparative physiological and transcriptomic analyses reveal the mechanisms of CO2 enrichment in promoting the growth and quality in Lactuca sativa
Source: PLoS One. 2023 Feb 3;18(2):e0278159. doi: 10.1371/journal.pone.0278159 (PMC9897578; doi:10.1371/journal.pone.0278159)
Supplement: Attachment — Submitted filename: response to reviewers.doc [file pone.0278159.s007.doc]

Dear Editor and Reviewers:

Thank you for your comments and for the reviewers’ comments concerning our manuscript entitled “Comparative physiological and transcriptomic analyses reveal the mechanisms of CO_2_ enrichment in promoting the growth and quality in *Lactuca sativa*”. Those comments are all valuable and very helpful for revising and improving our paper. We have checked the manuscript and revised it according to the comments. Changes were marked in colored text in the paper, Increased content was marked in red. Detailed responses to the comments of reviewer were attached. I hope that the revised manuscript is now suitable for publication.

Once again, thank you very much for your comments and suggestion.

Sincerely,

Hongxia Song

**Response to Reviewer 1:**

**Abstract**

1. Response to comment: (Page 2. in`… attention from all walks of …`the expression walks of life seems strange here, maybe authors can rewrite this sentence in a more scientific approach.)

Response: ‘from all walks of life’ had been deleted.

2. Response to comment: (Regarding the sentence `The rational utilization of CO_2_ by plants is an effective way to promote carbon neutrality`. How is possible that lettuce cultivation will promote carbon neutrality? Carbon neutrality achievement by plants is only possible when large carbon flux is occurring, such as CO_2_ flux in forest and other ecosystems. This information seems out of context here. In addition, authors need to decide what will be focus of the manuscript: (1) anthropogenic increase of atmospheric CO_2_ or (2) CO_2_ fertilization techniques in controlled environments to increase lettuce production. In the situation 1, authors need to put into account that productivity of plants in the future will also occur under elevated temperatures conditions and different rainfall patterns, therefore, the results obtained here cannot be extrapolated to future conditions since both, temperature and drought can negate or fully mitigate the benefits of a CO_2_ increase. In the situation 2, authors can focus only in CO_2_ fertilization under controlled conditions for a higher lettuce production, without taking into account the future increases in temperature, since plants are growing under controlled conditions (controlled temperature, CO_2_, etc). It seems that situation 2 is the more adequate approach for this manuscript and all sentences related to climate change and atmospheric CO_2_ increased by human activity are not necessary and should be removed.

Response: All sentences related to climate change and atmospheric CO_2_ increased by human activity had been removed.

3. Response to comment: (Along the manuscript authors used sentences such as `… effect of CO_2_ on lettuce…`. Authors are not testing the effects of CO_2_ on plants, but the effects of ELEVATED CO_2_ on plants. In phrases such as this one, it is important to include this information.)

Response: Full text revised as requested.

**Introduction**

1. Response to comment: (In general, introduction is well-written and concise. However, the introduction needs a hypothesis. Which plant responses authors expected according to the literature? In the discussion section, authors should clarify if the hypothesis was corroborated or not.)

Response: This part has been added in the Introduction and Discussion respectively.

**Material and methods**

1. Response to comment: (S1 Fig. Please, insert a subtitle in the supplementary figure.)

Response: S1 Fig. had been inserted a subtitle in the supplementary figure.

2. Response to comment: (Why authors decided to initiate the CO_2_ enrichment only after one week of transplanting? Is there any reason for this?)

Response: Plants could completely grow normally one week after transplanting, and rejuvenation period was ended.

3. Response to comment: (Please, change `release time` for `CO_2_ fumigation period`)

Response: `release time` had been revised.

4. Response to comment: (Why CO_2_ was not fumigated in rainy or snowy days? How many rainy/snowy days occurred during the experiment? Please, include both information in the manuscript.)

Response: In rainy and snowy days, the photosynthetic rate decreases, and higher concentrations of CO_2_ cannot be absorbed by plants. and there were 30 d for CO_2_ application in general. These are also included in the Materials and methods section.

5. Response to comment: (Please, describe in more details what `traditional management practices` means. I imagine that each lettuce color has a different management strategy. In addition, in different countries `traditional management practices` for lettuce cultivation will have different meanings.)

Response: This experiment adopts traditional soil cultivation, which is supplemented in the text. The specific management steps are cited in the literature [12].

6. Response to comment: (Which gas exchange parameters were measured? How light curves (light intensity steps) were conducted to obtain LSP and LCP parameters?)

Response: The measured gas exchange parameters include Gs and Ci. One leaf per plant was chosen for conducting the light curve, and the light intensity settings were 50, 100, 150, 200, 300, 400, 600, 800, 1000, 1200, 1400, 1600, 1800, 2000 and 2200 μmol•m^−2^•s^−1^, respectively. Related content has been supplemented in the Materials and methods section.

7. Response to comment: (Please, include the statistical methods used to analyze the data in the entire manuscript in the material and methods section. This information is crucial for data interpretation. Did authors used ANOVA? Student t tests?)

Response: Statistical methods had been supplemented.

**Results**

1. Response to comment: (Within each table we have lowercase and uppercase letters presumably indicating statistical differences between average values. However, we can`t find the meaning of these letters in the subtitle or methods section. Please, include this information in all subtitles.)

Response: The meaning of lowercase and uppercase letters had been supplemented.

2. Response to comment: (Page 10. The following sentence `The comprehensive analysis showed that the application of CO_2_ improved the light energy utilization rate to different extents and caused the plants to accumulate more photosynthetic products, which may partially explain the acceleration of leaf growth under CO_2_ enrichment of lettuce.` should be placed in the discussion section.)

Response: This sentence had been placed in the photosynthetic part of the discussion.

3. Response to comment: (Page 11. Again, sentences such as `Elevated CO_2_ in the environment will lead to changes in the content of various metabolites in plants, which will further affect their nutritional quality [16].` should be placed in other sections of the manuscript such as introduction or discussion.)

Response: This sentence has been placed in the Lettuce Growth and Quality section of the discussion.

4. Response to comment: (All subtitles can be improved with more details. In the current form, subtitles are too simple.)

Response: All subtitles are modified one by one.

**Discussion**

1. Response to comment: (It was a great idea to split the discussion in sub-sections. In general, discussion section is ok. However, especially in the subsection of photosynthesis, the discussion is too simple. Much more mechanistic discussion is possible using your data.)

Response: Discussion section had been added.
