# Supplementary material for: Comparative physiological and transcriptomic analyses reveal the mechanisms of CO2 enrichment in promoting the growth and quality in Lactuca sativa
Source: PLoS One. 2023 Feb 3;18(2):e0278159. doi: 10.1371/journal.pone.0278159 (PMC9897578; doi:10.1371/journal.pone.0278159)
Supplement: Attachment — Submitted filename: Response to Reviewers.doc [file pone.0278159.s008.doc]

Dear Editor and Reviewers:

Thank you for your comments and for the reviewers’ comments concerning our manuscript entitled “Comparative physiological and transcriptomic analyses reveal the mechanisms of CO_2_ enrichment in promoting the growth and quality in *Lactuca sativa*”. Those comments are all valuable and very helpful for revising and improving our paper. We have checked the manuscript and revised it according to the comments. Changes were marked in colored text in the paper, Increased content was marked in red. Detailed responses to the comments of reviewer were attached. I hope that the revised manuscript is now suitable for publication.

Once again, thank you very much for your comments and suggestion.

Sincerely,

Hongxia Song

**Response to Reviewer 2:**

Response to comment: (Authors have revised but still new references are missing Yr 2022- nil	Yr 2021~1)

Response: The discussion section had added content, using a total of 5 new references.

**Response to Reviewer 3:**

**1.** Response to comment: (Experiment details about CO_2_ application, what is the concentration of ambient CO_2_ and what is the concentration of elevated CO_2_?)

Response: The concentration of ambient CO_2_ is 400 μmol•mol^-1^ and the concentration of elevated CO_2_ is 800±50 μmol•mol^-1^, the CO_2_ concentration was monitored throughout the experiment. These were described in detail in lines 95-96 and lines 100-102.)

2. Response to comment: (Please clarify how the statistical significance was obtained, was the comparison between elevated CO_2_ and ambient CO_2_ done for each variable (from plant growth, photosynthesis, lettuce quality) and each variety or there are different grouping?)

Response: The comparison between elevated CO_2_ and ambient CO_2_ was done for the statistical significance for each variable (from plant growth, photosynthesis, lettuce quality) of each varietys. No significant difference analysis was performed between varieties. Relevant content has been provided and modified.

3. Response to comment: (Line 115-116 and 118-119: “One leaf per plant was chosen for conducting the light curve” and “Three healthy leaves were selected for each measurement”. Please provide details for how these leaf were selected, were they consistent between different plants?)

Response: A total of three plants were selected for each treatment of each variety, and one leaf was selected per plant for determination, and then the average value was calculated. The leaf positions of each variety and treatment were the same. Relevant content has been provided and modified.

4. Response to comment: (Line 133-136: It seems that there is only one replicate used for RNA-seq from S6, S16, S24 varieties. Please clarify this in the methods.)

Response: There is only one replicate used for RNA-seq from S6, S16, S24 varieties. This had been clarified and supplemented in the methods.

5. Response to comment: (Line 140-147: Please provide additional information for 1) how the RNA-seq data was pre-processed; 2) how the RNA-seq reads were mapped (tool and genome used); 3) how DEGs were identified (which tool was used); 4) whether DEGs were identified for each lettuce variety separately or all together; and 5) how functional enrichment was performed for both KEGG and GO (what tools were used). Reference Sun et al. doesn’t provide these details.)

Response: All of additional information had been provided.

6. Response to comment: ( Line 177: 1) Please clarify how leaf width, plant height and width were defined in the current study; 2) Please reformat Table 1 to have a clear separation between S6, S16 and S24 groups.)

Response: Leaf width, plant height and width were defined in methods. Table 1 has been reformatted.

7. Response to comment: (The authors mentioned multiple RNA-seq studies from line 64-74 about the effect of elevated CO_2_ in different species. Did the authors compare the results from current study to the results from these previous studies to see if there are any genes/pathways being differentially regulated by elevated CO_2_ regardless of species or these species might have very distinct response to the elevated CO_2_?)

Response: C3 and C4 crops respond differently to CO_2_. Our team has been working on CO_2_ enrichment research of vegetable for many years, since most vegetables are C3 plants with short growth cycle and are sensitive to carbon dioxide, they all show promotion effect at present. This was also reflected in the discussion.

8. Response to comment: (Please provide figures with higher resolution.)

Response: The clarity of the figures has been improved.

9. Response to comment: (Line 198: Please justify why there are uppercase and lowercase significance letters. Do they represent different grouping?)

Response: Difference analysis was not performed between varieties, but significant difference analysis was performed only between controls and treatments of the same variety. These have been added in the note.

10. Response to comment: (Data used in the current study doesn’t seem to be accessible accession number PRJNA859388 (<http://www.ncbi.nlm.nih.gov/bioproject/859388>).

Response: The current link will not be released until the article is published, if necessary, you can use the following address to view. ( https://dataview.ncbi.nlm.nih.gov/object/PRJNA859388?reviewer=3q9f1u02qig63u5jeclng0tmai)
