# Supplementary material for: Comparative physiological and transcriptomic analyses reveal the mechanisms of CO2 enrichment in promoting the growth and quality in Lactuca sativa
Source: PLoS One. 2023 Feb 3;18(2):e0278159. doi: 10.1371/journal.pone.0278159 (PMC9897578; doi:10.1371/journal.pone.0278159)
Supplement: Attachment — Submitted filename: Response to reviewers.doc [file pone.0278159.s009.doc]

Dear Editor and Reviewers:

Thank you for your comments and for the reviewers’ comments concerning our manuscript entitled “Comparative physiological and transcriptomic analyses reveal the mechanisms of CO_2_ enrichment in promoting the growth and quality in *Lactuca sativa*”. Those comments are all valuable and very helpful for revising and improving our paper. We have checked the manuscript and revised it according to the comments. Increased content was marked in red. Detailed responses to the comments of reviewer were attached. I hope that the revised manuscript is now suitable for publication.

The study was in part supported by the Shanxi Province Key Research and Development Program Key Projects under Grant (201703D211001-04-01), funded by the Shanxi Province Key Research and Development Program Key Projects under Grant (201903D211011). The funders had role in publication.

Once again, thank you very much for your comments and suggestion.

Sincerely,

Hongxia Song

**Response to Reviewer 3:**

Response to comment: (The authors addressed most of the initial comments. However, the use of only one replicate for RNA-seq is lower than the minimum requirement of three replicates, which will dramatically reduce the statistical power of detecting DEGs. One way the authors could consider is to use S6, S16, S24 varieties as biological replicates to investigate the effect of elevated CO_2_.)

Thank you very much for your advice. In fact, we also studied three species（S6, S16, S24 varieties）as three biological replicates. Moreover, it has been supplemented in the manuscript.
